# Farnesiferol C Induces Apoptosis in Chronic Myelogenous Leukemia Cells as an Imatinib Sensitizer via Caspase Activation and HDAC (Histone Deacetylase) Inactivation

**DOI:** 10.3390/ijms20225535

**Published:** 2019-11-06

**Authors:** Ji Hoon Jung, Ji Eon Park, Deok Yong Sim, Eunji Im, Woon Yi Park, Duckgue Lee, Bum-Sang Shim, Sung-Hoon Kim

**Affiliations:** College of Korean Medicine, Kyung Hee University, Seoul 02447, Korea; johnsperfume@gmail.com (J.H.J.); wdnk77@naver.com (J.E.P.); simdy0821@naver.com (D.Y.S.); ji4137@naver.com (E.I.); wy1319@naver.com (W.Y.P.); tomyoo27@gmail.com (D.L.); eshimbs@khu.ac.kr (B.-S.S.)

**Keywords:** farnesiferol C, imatinib, CML, HDAC, apoptosis

## Abstract

Herein the underlying apoptotic mechanism of Farnesiferol C (FC) derived from *Ferula assafoetida* was elucidated in chronic myelogenous leukemia (CML) K562 and KBM5 cells. FC showed significant cytotoxicity in K562 and KBM5 cells, more so than in U937 and UL-60 acute myeloid leukemia (AML) cells. Cleaved PARP and caspase 9/3 attenuated the expression of Bcl2 and induced G1 arrest in K562 and KBM5 cells. Also, FC effectively abrogated the expression of cell cycle related proteins, such as: Cyclin D1, Cyclin E, Cyclin B1 in K562, and KBM5 cells, but caspase 3 inhibitor Z-DEVD-FMK rescued the cleavages of caspase 3 and PARP induced by FC in K562 cells. Of note, FC decreased histone deacetylase 1 (HDAC1) and HDAC2, and enhanced histone H3 acetylation K18 (Ac-H3K18) in K562 and KBM5 cells. Furthermore, combination of FC and Imatinib enhanced the apoptotic effect of Imatinib as a potent Imatinib sensitizer in K562 cells. Overall, our findings provide scientific evidence that inactivation of HDAC and caspase activation mediate FC induced apoptosis in CML cells.

## 1. Introduction

Among bone marrow or blood cancers, leukemia with the abnormal proliferation feature of white blood cells is generally classified into myelogenous leukemia and lymphoblastic leukemia [1]. Chronic myeloid leukemia (CML) is a myeloproliferative disorder of transformed hematopoietic stem cells by the Philadelphia chromosome or the Philadelphia translocation (Ph) of chromosomes 9 and 22 with constitutively activated Bcr-abl tyrosine kinase [2]. Although various chemotherapeutical agents have been developed such as Imatinib mesylate (Gleevec^®^, Novartis), Busulfan (Myleran^®^, Busilvex), or Hydroxyurea (Hydrea^®^, Bristol-Myers Squibb Pharmaceuticals) for the treatment of CML, their therapeutic efficacies have been limited due to their side effects, such as lethargy, fluid retention, thrombocytopenia, nausea, and diarrhea [3,4].

Recently, anticancer agents from natural products such as decursin [5], tanshinone IIA [6], and curcumin [7] are attractive in CML cells due to their lesser toxicity and potent synergy with conventional anticancer drugs.

Histone acetylation is regarded as a dynamic process regulated by the antagonistic actions of two large families of enzymes, such as histone deacetylases (HDACs) and histone acetyltransferases (HATs) [8]. Histone deacetylases (HDACs) function to remove acetyl group from an N-acetyl lysine amino acid on a histone. Usually HDACs are overexpressed in several cancers and are known to be involved in cell survival, inflammation, proliferation, angiogenesis, and immunity [9]. Hence, HDAC inhibitors have also been considered potent agents for adjuvant therapy and cancer treatment [10].

Farnesiferol C (FC) derived from *Ferula asafoetida* species is a polycyclic aromatic compound containing a 1-benzopyran moiety with a ketone group at the C2 carbon atom. Though FC is known to have antileishmanial [11], antiangiogenic [12], and apoptotic effects [13,14,15,16], to date its underlying antitumor mechanisms still remain unclear in CML cells. Hence, in the current study, apoptotic mechanism of FC and its potential as an Imanitib sensitizer for combination therapy were evaluated in K562 and KBM5 CML cells.

## 2. Results

### 2.1. FC (Farnesiferol C) Induces Significant Cytotoxicity in K562 and KBM5 Cells.

**Figure 1:**
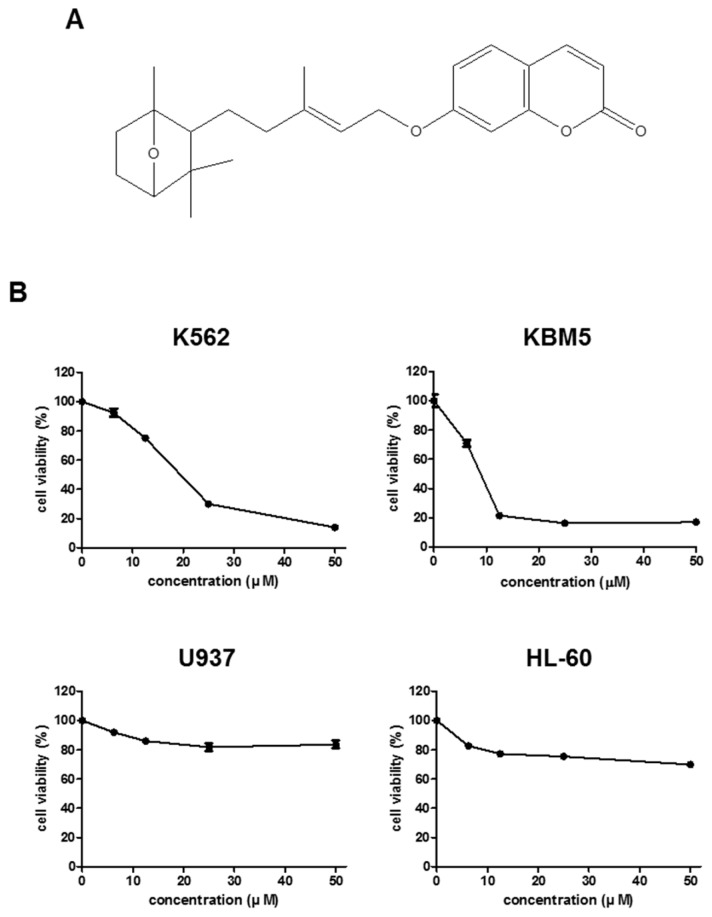
Cytotoxic effect of Farnesiferol C (FC) in leukemia. (**A**) Chemical structure of FC. (**B**) K562, KBM5, U937, and HL-60 cells were seeded into 96 well microplates and treated with various concentrations (10, 20, 30, 40, and 50 µM) of FC for 24 h. Cell viability was measured by 3-(4,5-Dimethylthiazol-2-yl)-2,5-diphenyltetrazolium bromide (MTT) assay.

To confirm the cytotoxicity of FC (Figure 1A), a cell viability assay was conducted in K562, KBM5, U937, and HL-60 cells by an MTT assay. Here, FC significantly reduced the viability of K562 and KBM5 cells (CML) in a concentration dependent fashion, better than in U937 and HL-60 AML cells (Figure 1B).

### 2.2. FC Regulates Apoptosis Related Proteins and Induces G1 Arrest in CML Cells.

**Figure 2:**
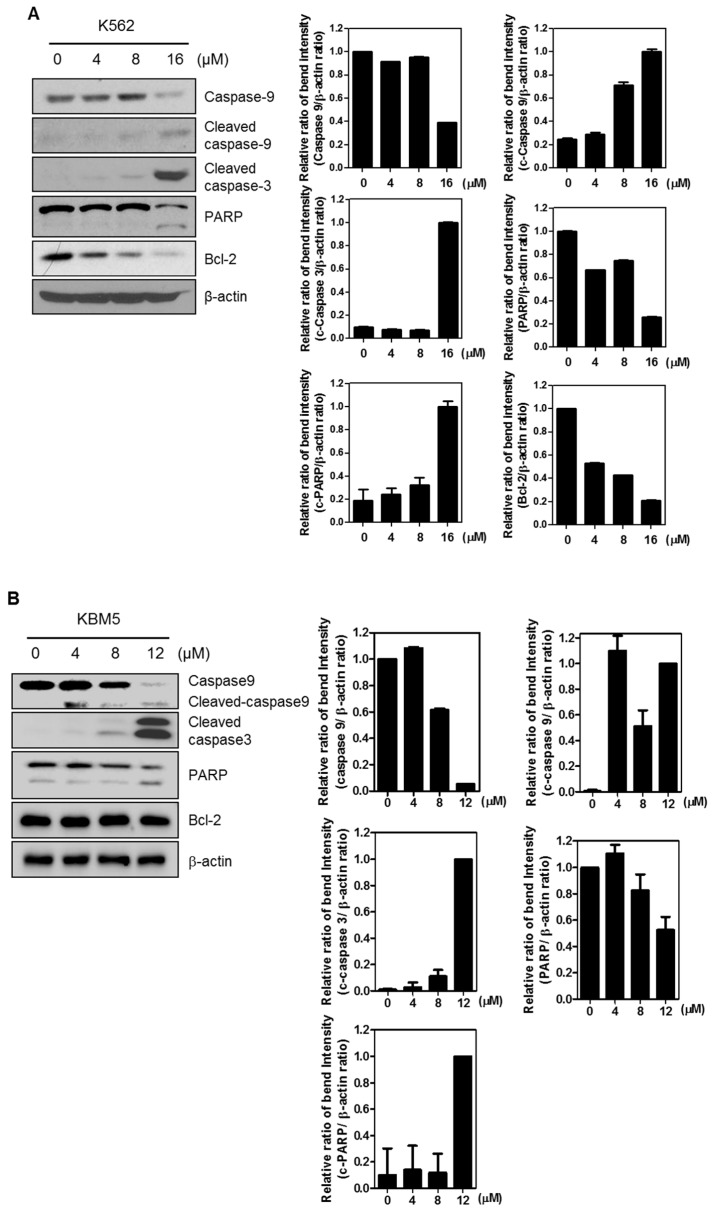

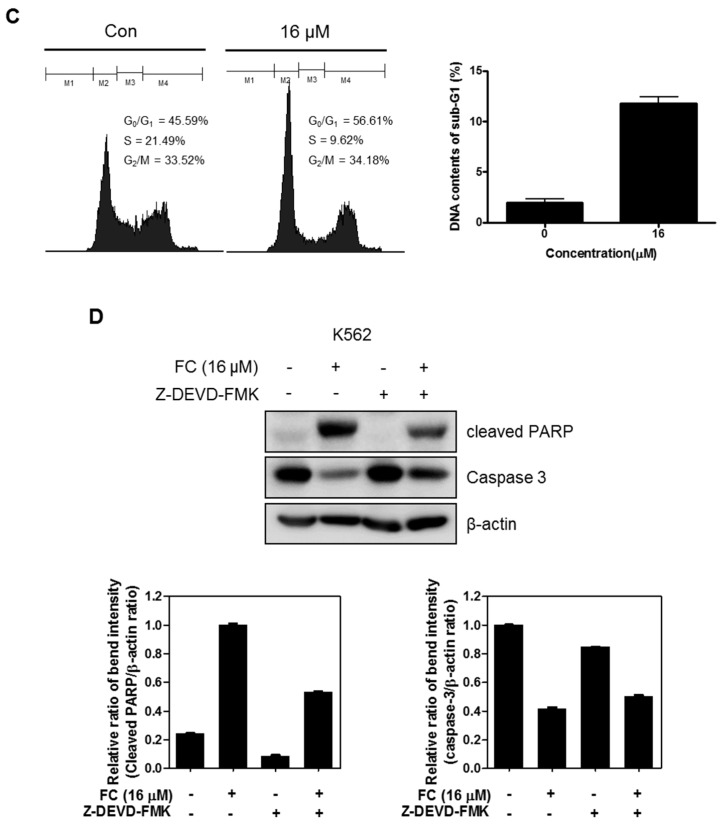
FC regulates apoptosis-related proteins and increases G1 arrest in K562 cells. (**A**,**B**) Effect of FC on procaspase9, cleaved caspase9, cleaved caspase3, PARP, and Bcl-2 in a concentration dependent fashion in K562 and KBM5 cells. (**C**) Effect of FC (16 µM) on G1 arrest in K562 cells by Fluorescence-activated cell sorting (FACS) analysis. (**D**) Effect of caspase 3 inhibitor, Benzyloxycarbonyl-Asp(OMe)-Glu(OMe)-Val-Asp(OMe)-fluoromethylketone (Z-DEVD-FMK), on FC induced apoptosis in K562 cells.

To examine whether the cytotoxic effect of FC is associated with apoptosis, the effect of FC on apoptosis related genes was evaluated in K562 or KBM5 cells. FC induced the cleavages of PARP, caspase-9, and caspase-3, and decreased the expression of Bcl-2 in K562 and KBM5 cells (Figure 2A,B). Additionally, as shown in Figure 2C, FC increased sub-G1 population in K562 cells. Conversely, caspase 3 inhibitor Z-DEVD-FMK rescued cleavages of caspase 3 and PARP in K562 cells (Figure 2D).

### 2.3. FC Regulates Cell Cycle Related Proteins

**Figure 3:**
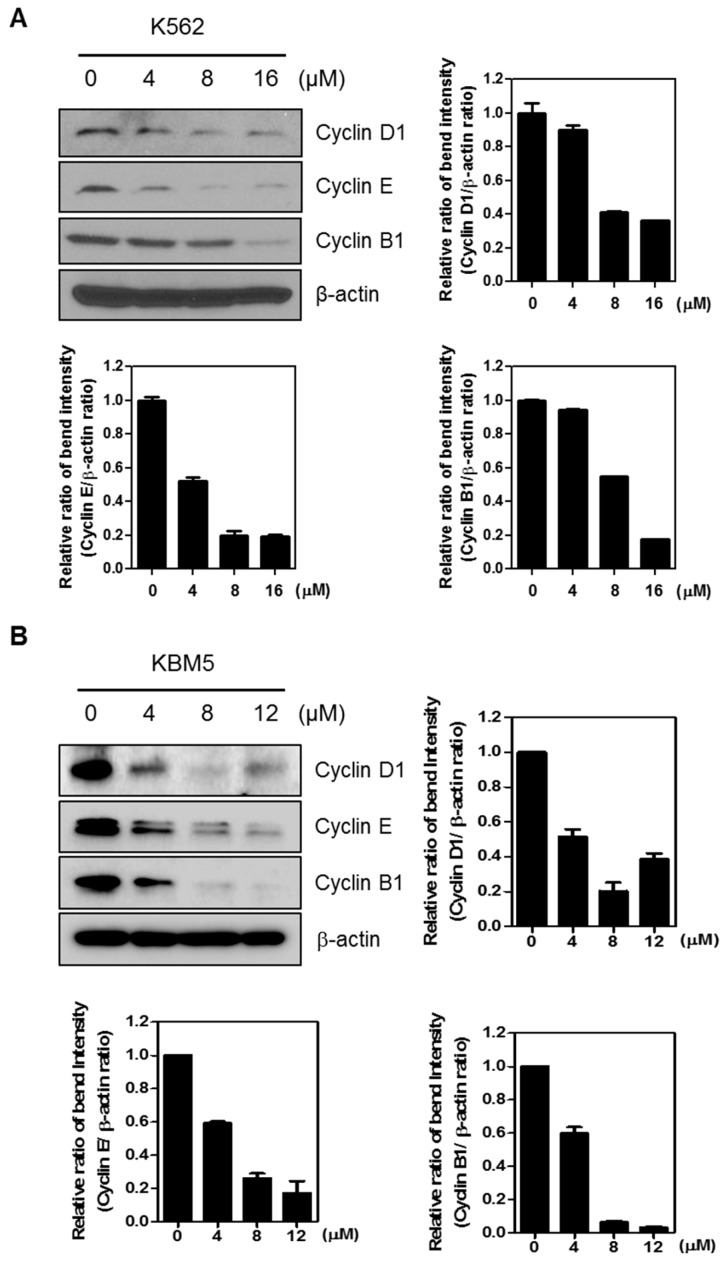
FC regulates cell cycle related proteins in (**A**) K562 and (**B**) KBM5 cells. K562 and KBM5 cells were treated with FC (4, 8, or 16 µM) for 24 h. Cell extracts were prepared and subjected to Western blotting with Cyclin D1, E, and B1 antibodies. β-actin was used as an internal control.

It is well known that FC induces cell cycle arrest in breast cancer cells [17]. To investigate whether FC regulates cell cycle proteins, Western blotting was performed in K562 and KBM5 cells. As shown in Figure 3, FC inhibited the expression of cyclin D1, cyclin E, and cyclin B1.

### 2.4. FC Regulates HDAC (Histone Deacetylase) 1 and 2 through Acetylation H3 (K18) in CML Cells.

**Figure 4:**
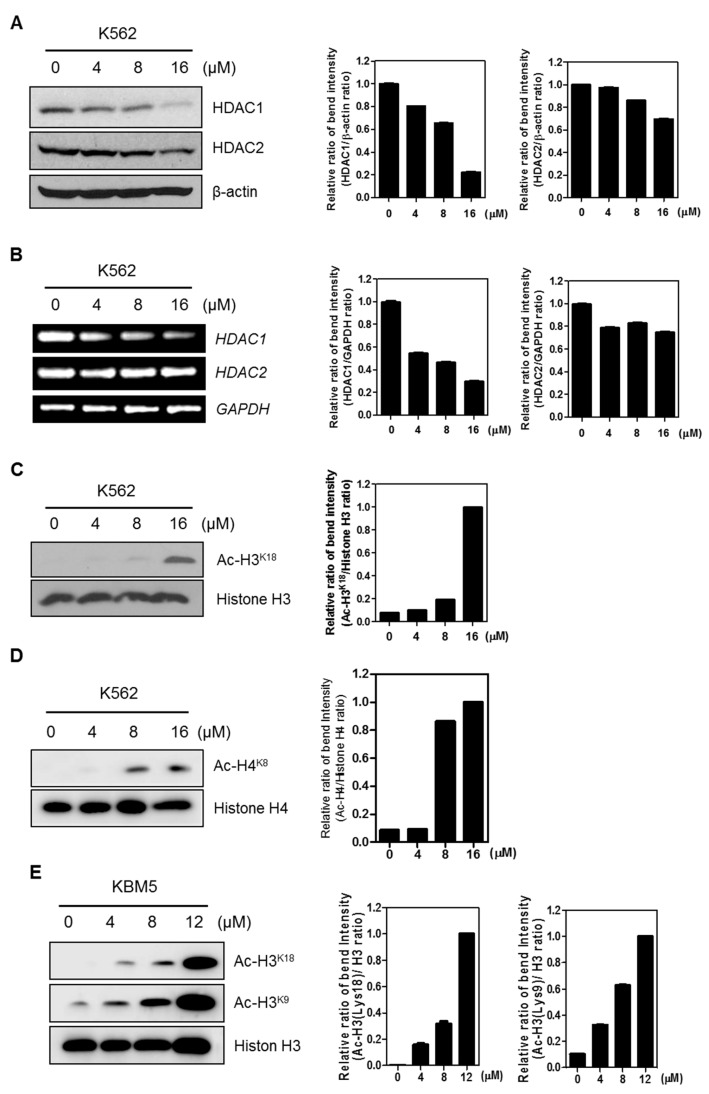
FC inhibits the expression of HDAC1 and 2 at protein and mRNA levels, and induces acetylation of H3^K9^, H3^K18^, and H4^K8^ in CML cells. (**A**) Effect of FC on HDAC1 and HDAC2 in a concentration dependent manner in K562 cells. Cell extracts were prepared and subjected to Western blotting with antibodies of HDAC1 and 2. β-actin was used as an internal control. (**B**) Effect of FC on HDAC1 and HDAC2 in a concentration dependent fashion in K562 cells at mRNA level. Isolated RNAs were subjected to RT-PCR for HDAC1 and 2. Glyceraldehyde 3-phosphate dehydrogenase (GAPDH) was used as an internal control. (**C**,**D**) Effect of FC on acetylation H3 (K18) or H4 (K8) in K562 cells. Cell extracts were prepared and subjected to Western blotting with acetylation H3 (K18) or H4 (K8) antibodies. Histone H3 or H4 were used as an internal control. (**E**) Effect of FC on acetylation of H3 (K18) or H3 (K9) in KBM5 cells.

Histone acetylation is regulated by the balance between histone deacetylases (HDACs) and histone acetyltransferases (HATs). Among 18 HDACs, HDAC2 and HDAC1 are included in Class 1 of HDACs [18]. Herein, FC attenuated the protein expression of HDAC1 and HDAC2 (Figure 4A) and also reduced the mRNA expression of HDAC2 and HDAC1 (Figure 4B) in K562 cells. Furthermore, FC upregulated the expression of histone H3 acetylation K18 (Ac-H3^K18^) (Figure 4C) and histone H4 acetylation K8 (Ac-H4^K8^) (Figure 4D) in K562 cells. Similarly, FC upregulated the expression of histone H3 acetylation K18 (Ac-H3^K18^) and H3 acetylation K18 (Ac-H3^K9^) in KBM5 cells (Figure 4E).

### 2.5. The Role of HDAC1 in FC Induced Apoptosis in K562 Cells.

**Figure 5:**
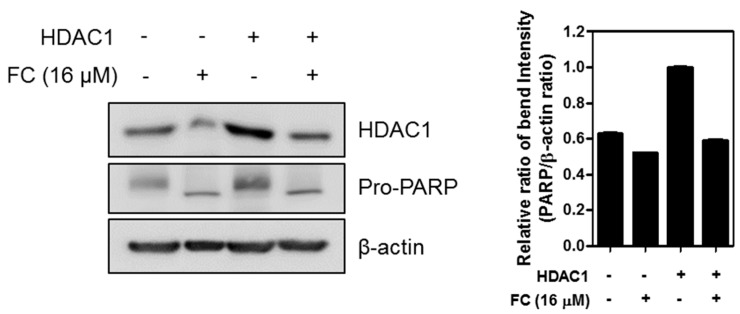
The role of HDAC1 in FC induced apoptosis in K562 cells. K562 cells were transfected with HDAC1 plasmids and treated with or without FC, respectively, and then subjected to Western blotting with antibodies of HDAC1 and PARP. β-actin was used as an internal control.

Next, the role of HDAC1 was examined in FC induced apoptosis. The effect of HDAC1 overexpression was tested on pro-PARP expression in K562 cells in the presence or absence of FC. As shown in Figure 5, overexpression of HDAC1 weakly reduced the apoptotic ability of FC to attenuate pro-PARP expression compared to the FC alone control, which may be due to transfection efficiency in K562 suspension cells.

### 2.6. FC Sensitizes K562 Cells to Imatinib Induced Apoptosis.

**Figure 6:**
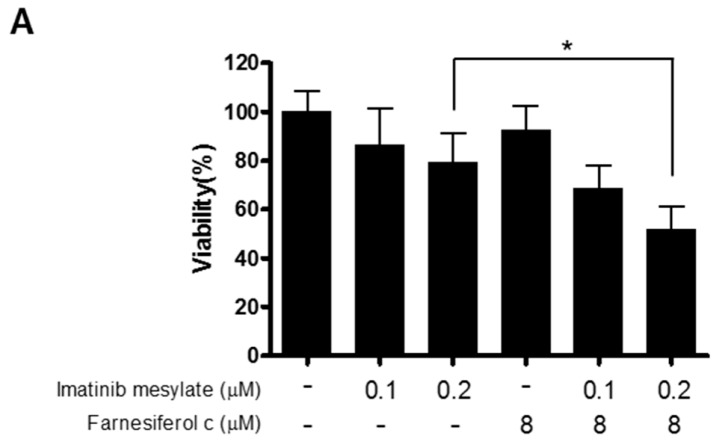

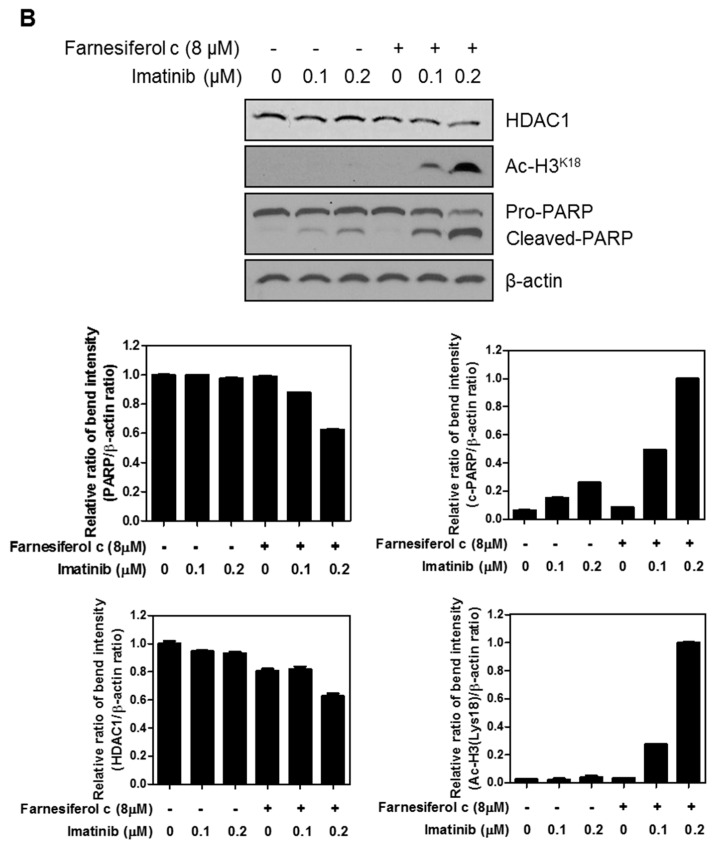
Combinatorial effect of FC and Imatinib in K562 cells (**A**) K562 cells were distributed into 96 well microplates and were exposed to Imatinib (0, 0.1, 0.2 µM) and/or FC (8 µM) for 24 h. Cell viability was evaluated by an MTT assay. *, *p* value < 0.05 between Imatinib alone and FC and Imatinib. (**B**) Effect of FC on HDAC1, acetylation H3 (K18), and PARP in a concentration dependent fashion in K562 cells. Cell extracts were prepared and subjected to Western blotting was conducted with cell extracts by using HDAC1 and 2 antibodies. β-actin was used as an internal control.

To confirm the potential of FC as an Imatinib sensitizer, the combinatorial effect of FC and Imatinib was evaluated in K562 cells. As shown in Figure 6A, cotreatment of FC and Imatinib significantly reduced viability of K562 cells compared to Imatinib alone control (Figure 6A). Similarly, cotreatment of FC and Imatinib attenuated the expression of HDAC1, upregulated Ac-H3K18, and cleaved PARP in K562 cells (Figure 6B).

## 3. Discussion

In the current project, the underlying apoptotic mechanism of FC and its potential of Imanitib sensitizer for combinatorial therapy were explored in CML cells. Herein, FC exerted significant cytotoxicity in K562 and KBM5 CML cells better than in U937 and HL-60 AML cells, implying FC may be more susceptible to CML cells with less blast cells than AML cells with more blast cells. It also suggests the better antitumor effect of FC in CML cells, since AML cells have more than 20% of blast cells for proliferation and differentiation than CML cells. In addition, FC was a known nontoxic in normal mesenchymal stem cells [16].

Generally, apoptosis is induced via intrinsic (mitochondrial) or extrinsic (cell death) dependent pathway with features of chromatin condensation, nuclear fragmentation, blebbing, cell shrinkage, and global mRNA decay [19,20]. Here, FC induced cleavages of PARP and caspase 9/3, attenuated the expression of Bcl2, one of antiapoptotic proteins [21] in K562 and KBM5 cells, and conversely caspase 3 inhibitor Z-DEVD-FMK rescued cleavages of PARP and caspase 3 induced by FC in K562 cells, indicating apoptotic effect FC via mitochondrial dependent apoptotic pathway.

It is well documented that cell cycle consists of four distinguishable phases such as G1, S (synthesis), G2 (interphase), and M (mitosis) phases for cell division and duplication, and inhibition of cell cycle is considered a potent strategy for cancer therapy [22,23]. Here FC induced G1 arrest in K562 cells and effectively inhibited the expression of Cyclin D1, Cyclin E, Cyclin B1 that are related to G1-S phase transition [24], demonstrating G1 arrest effect of FC.

Emerging evidences reveal that histone acetylation modulated by histone deacetylases (HDACs) and histone acetyltransferases (HATs) is critically involved in cancer progression [10,25,26]. Here FC decreased histone deacetylase 1 (HDAC1) and HDAC2, and induced acetylation of H3^K9^, H3^K18^, and H4^K8^ in K562 and KBM5 cells, implying the critical role of HDAC inhibition in FC induced apoptosis in CML cells. Similarly, FC was known to induce apoptosis via modulation of c-Myc and in non-small-cell lung cancers [13] and also exerted antitumor and antiangiogenic effects by multiple targets of VEGFR1 or VEGFR2 signaling [12].

Though Imatinib has been extensively used for treatment of CML for years, recently combination therapy with low dose of Imatinib is attractive to reduce side effects due to its toxicity in normal cells [27,28]. In the same line, FC sensitized K562 cells to Imatinib induced apoptosis by inhibition of HDAC1, activation of Ac-H3K18, and PARP cleavage in K562 cells, strongly indicating combinatorial therapy potential of FC with Imatinib.

**Figure 7:**
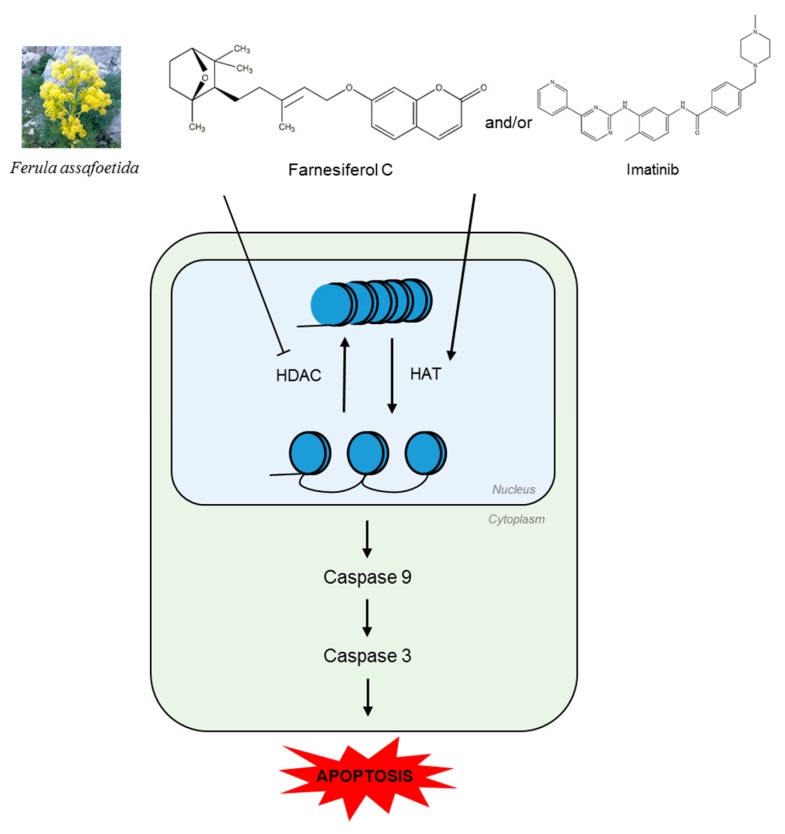
Graphical abstract on the apoptotic mechanism of FC in chronic myelogenous leukemia cells as an Imatinib sensitizer via caspase 3 activation and HDAC inactivation. Blue circle indicates histone protein.

In summary, FC showed significant cytotoxicity, cleaved PARP, and caspase 9/3, attenuated the expression of Bcl2, Cyclin D1, Cyclin E, Cyclin B1, and induced G1 arrest in K562 and/or KBM5 cells. Also, caspase 3 inhibitor Z-DEVD-FMK rescued cleavages of caspase 3 and PARP induced by FC in K562 cells. Furthermore, FC decreased HDAC1 and HDAC2, enhanced Ac-H3^K18^ in K562 cells, and sensitized K562 cells to Imatinib induced apoptosis. Taken together, our findings provide insight that inactivation of HDAC and caspase3 activation are critically involved in FC induced apoptosis in CML cells (Figure 7).

## 4. Materials and Methods

### 4.1. Chemicals and Reagents

FC (Figure 1A) was isolated and identified from *Ferula assafoetida* as previously described [12]. 3-(4,5-dimethylthiazol-2-yl)-2,5-diphenyltetrazolium bromide (MTT), Bcl-2, and β-actin were purchased from Sigma-Aldrich (Sigma-Aldrich, St. Louis, MO, USA). Also, specific antibodies for Cyclin D1, Cyclin E, Cyclin B1 were bought from Santa Cruz Biotechnology (Santa Cruz Biotechnology, Dallas, TX, USA). PARP, HDAC1, and HDAC2 were purchased from Cell Signaling (Cell signaling Technology, Danvers, MA, USA) for Western blot analysis.

### 4.2. Cell Culture

Human K562 (ATCC^®^ CCL-243™) myeloid leukemia cells (CML), KBM5 CML cells (RRID: CVCL_0373), U937 (ATCC^®^ CRL1593.2™) acute myeloid leukemia (AML) cells, and HL-60 (ATCC® CCL-240™) AML cells were maintained in RPMI1640 medium supplemented with 2 μM L-glutamine, penicillin/streptomycin, and 10% fetal bovine serum (FBS).

### 4.3. MTT (3-(4,5-Dimethylthiazol-2-yl)-2,5-Diphenyltetrazolium Bromide) Assay

Cytotoxic effect of FC and/or Imatinib in K562 cells, KBM5, U937, and HL-60 cells was examined by using MTT assay according to the method shown in Jung et al.’s paper [6]. The cells (1 × 10^4^ cells per well) were exposed to various concentrations of FC (10, 20, 30, 40, and 50 µM) and/or Imatinib (0, 0.1, 0.2 µM) for 24 h, then with MTT (1 mg/mL) for 2 h, and exposed to DMSO for 20 min. Thereafter, optical density was measured with a microplate reader (Molecular Devices Co., Silicon Valley, CA, USA) at 570 nm. Cell viability was evaluated as a percentage of viable cells in FC and/or Imatinib treated group vs. untreated control.

### 4.4. Cell Cycle Analysis

Based on cell cycle method shown in Yun et al.’s paper [29], cell cycle analysis was conducted in K562 cells treated with or without FC by propidium iodide (PI) staining. Cell cycle distributions were calculated by FACS Calibur (Becton Dickinson, Franklin Lakes, NJ, USA) by using the Cell Quest program (Becton Dickinson).

### 4.5. RNA Isolation and Real Time Polymerase Chain Reaction (RT-PCR)

Based on RT-PCR method shown in Jung et al.’s paper [12], total RNAs from K562 cells treated with FC were isolated, amplified, and quantified by using Superscript One Step RT-PCR kit (Invitrogen, Carsbad, CA, USA) with Platinum Taq polymerase. Primers sequences were synthesized by Bioneer (Daejeon, Korea) with the following sequences: hGAPDH-forward 5′-CCA CTC CTC CAC CTT TGA CA-3′; reverse-5′-ACC CTG TTG CTG TAG CCA-3′, HDAC1-forward: 5′-GAC TCT GAG ACA GTG CTT CGA TGA-3′; reverse-5′-CCA TGA GGC CCA ACT TCC T-3′.

### 4.6. Western Blotting

K562 or KBM5 cells were exposed to FC and/or Imanitib for 24 h and subjected to Western blotting based on method shown in Lee et al.’s [12]. The protein samples were separated and transferred to nitrocellulose membranes. Membranes were incubated with primary antibodies of HDAC1, HDAC2, Ac-H3^K9^, Ac-H3^K18^, and Ac-H4^K8^, cleaved PARP, PARP, caspase-9, caspase 3, and finally, incubated with HRP-conjugated secondary antibody (1:2000). The expression was visualized by using ECL Western blotting detection reagent (GE Healthcare, Amersham, UK).

### 4.7. Transfection Assay

K562 cells were transfected with control vector or HDAC1 plasmid with Lipofectamine 2000 and Interferin™ transfection reagent (Polyplus-transfection Inc., New York, NY, USA). The mixtures of HDAC1 plasmid and transfection reagent were together incubated for 10 min and then the cells were incubated at 37 °C for 36 h before exposure to FC (16 µM for 24 h).

### 4.8. Statistical Analysis

The data stand for means ± SD from at least three independent experiments. Student’s t-test was used for two group comparison and the one-way analysis of variance (ANOVA), followed by a Turkey post-hoc test for multi-group comparison by using GraphPad Prism software (Version 5.0, California, CA, USA). The statistically significance was accepted, only when the difference p-value between groups was less than 0.05.

## Abbreviations

FC	Farnesiferol C
PARP	Poly (ADP-ribose) polymerase
Caspase	Cysteine aspartyl-specific protease
Bcl-2	B-cell lymphoma 2
HDAC	Histone deacetylase
